# Identification of a novel fiber shaft structural motif and overexpression of key transcripts elucidated in human adenovirus D 10

**DOI:** 10.1371/journal.ppat.1014182

**Published:** 2026-04-28

**Authors:** Rosie M. Mundy, Kasim Waraich, Emily A. Bates, Pierre J. Rizkallah, Alexander T. Baker, Mark T. Young, Edward Morris, Paula C. A. da Fonseca, Carly M. Bliss, David Matthews, David Bhella, Alan L. Parker

**Affiliations:** 1 Division of Cancer and Genetics, Cardiff University School of Medicine, Cardiff, United Kingdom; 2 MRC – University of Glasgow Centre for Virus Research, Glasgow, United Kingdom; 3 School of Biochemistry, University Walk, University of Bristol, Bristol, United Kingdom; 4 Division of Infection and Immunity, Cardiff University School of Medicine, Cardiff, United Kingdom; 5 School of Biosciences, Cardiff University, Cardiff, United Kingdom; 6 School of Molecular Biosciences, University of Glasgow, Glasgow, United Kingdom; 7 Wales Applied Virology Unit, Cardiff University School of Medicine, Cardiff, United Kingdom; 8 Department of Cellular and Molecular Medicine, School of Medical Sciences University Walk, University of Bristol, Bristol, United Kingdom; The Children's Hospital of Philadelphia, UNITED STATES OF AMERICA

## Abstract

Adenoviruses are widely used as vectors for subunit vaccines and oncolytic therapies. Efficient vectors must infect target cells and deliver therapeutic transgenes at high levels. Species D adenoviruses, such as human adenovirus type 10 (HAdV-D10), are promising candidates due to low seroprevalence in humans. Here, we present the cryo-electron microscopy structure of the HAdV-D10 capsid alongside transcriptomic profiling of infected cells to inform vector development. The fiber shaft, essential for cell entry, was resolved at 10 Å, revealing a previously uncharacterized ‘umbrella’ motif. Viral transcript analysis using an ORF-centric pipeline uncovered key differences from HAdV-C5, including abundant expression of a transcript encoding a protein equivalent to mature protein VII. These findings highlight the importance of detailed vector characterization prior to clinical translation and support the advancement of HAdV-D10 as a next-generation platform for gene delivery and vaccine development.

## Author summary

Adenoviruses cause infections with pathologies ranging from cold like respiratory symptoms to gastroenteritis and conjunctivitis. In addition to research on adenovirus pathology, substantial research has focused on adenoviruses as gene delivery vectors, with such therapeutics seeing widespread use during the COVID-19 pandemic. More recently there is interest in the use of adenovirus vectors for cancer treatment. Oncolytic adenovirus vectors’ tropisms can be adapted to selectively infect cancerous cells through targeting of upregulated surface exposed cancer markers such epidermal growth factor receptor. Infection results in cell lysis, releasing cancer antigens, whilst also delivering a genome encoded anti-tumour ‘payload’ in a potent cancer treatment associated with milder side-effects than chemotherapy. Here we report the cryo-EM structure and transcriptomic analysis of human adenovirus D 10, a low seroprevalence serotype with the aim of providing preliminary data with a focus on capsid structure, with the intention to later defining capsid-host interactions, and transcriptomics to ensure the serotype will efficiently and safely deliver a genome encoded cancer payload. Key findings are a novel fiber shaft motif and abundant transcription of the viral encoded DNA binding protein VII. Our study highlights that not all adenoviruses are the same and should be researched prior to use as vectors.

## Introduction

Human adenoviruses (HAdV) are double stranded DNA viruses with non-enveloped icosahedral capsids [1]. HAdV are split across species A-G based on serology, sequence identity and pathogenicity [2]. Species D (HAdV-D) is phylogenetically the largest species of HAdV as a result of recombination events giving rise to new adenoviruses serotypes [3]. HAdV-D viruses often cause minor illness, including ocular infections [1]. Adenovirus 10 (HAdV-D10) was isolated from the eyes of conjunctivitis patients with clinical symptoms distinct from other conjunctivitis with adenovirus etiology: increased ocular pressure and the presence of pseudomembranes, thin fibrin-rich films that form on the conjunctiva as a consequence of inflammatory secretions [4–7]. HAdV-D10 has low seroprevalence across human populations making it a compelling candidate for development as a therapeutic vector [8].

Current therapies utilising adenovirus vectors rely heavily on species C adenovirus 5 (HAdV-C5) which has high seroprevalence across multiple populations, limiting existing therapies’ efficacy as they are neutralized by patients’ immune responses [9]. This makes vectors based on alternative, low seroprevalence serotypes more attractive as they are less likely to be recognised and neutralised by the immune system and therefore more likely to reach the target tissue [9]. HAdV-D10 has the added benefit of low affinity receptor interaction with previously identified adenovirus receptors including Coxsackie and Adenovirus Receptor (CAR) and sialic acid, and no interaction with CD46 making it a potential ‘blank slate’ vector candidate, amenable to tissue specific targeting [8].

Cryo-electron microscopy (cryo-EM) has been used to determine the whole capsid structure of multiple adenoviruses including HAdV-C5, adenovirus 26 (HAdV-D26), adenovirus 41 (HAdV-F41) and the simian adenovirus ChAdOx1 derived from species E Y25 isolate, the platform vector for the ChAdOx nCoV-19 vaccine [10–14]. Visualizing whole virus capsids during development of therapeutic vectors has previously helped with understanding off-target effects of viral vaccines and therefore may help guide engineering strategies ahead of clinical translation [14].

Adenoviruses have a conserved icosahedral capsid structure, consisting of three major proteins: hexon, penton base and fiber proteins. Hexon protein, the most abundant of the capsid, forms 240 trimeric capsomeres making up the facets of the icosahedron [11,12]. Hexon protein has seven hypervariable regions (HVRs), mostly loops, containing the most inter and intra species sequence dissimilarity [12,15].

A pentamer of penton base proteins is found at each of the twelve vertices. Penton structure contains an RGD motif which is essential for engagement with host integrins to trigger virion internalisation [16]. Penton capsomere also anchors the fiber to the capsid. The fiber is trimeric, with the shaft extending away from the capsid and terminating in the globular knob domain which acts as a high-affinity tether by interacting with host cellular receptors such as CAR, CD46 and sialic acid (SA) [17–19].

Minor proteins, protein IIIa, IX, VIII and VI, play a structural role in the capsid alongside the major proteins. Core proteins V, VII and Mu (also known as protein X) interact with and bind DNA in the capsid. Precursor proteins pVI, pIIIa, pTP, pVII and pMu are processed by the virally encoded protease from precursor to their mature forms [20]. The capsid itself possesses pseudo-T = 25 icosahedral symmetry [21]. Furthermore, the fiber protein located at each five-fold vertex is trimeric and has a symmetry mismatched interaction with the pentameric penton capsomere, making resolution by cryo-EM challenging. There has been one attempt to resolve the fiber structure to low resolution, but none have successfully achieved a high resolution structure [11].

We pair the structure of HAdV-D10 with transcriptomic investigations to add to the body of knowledge around HAdV-D10. We use an open reading frame (ORF)-centric analysis pipeline of long read direct RNA-seq (dRNA-seq) data to cover the complex transcriptome of HAdV-D10 [22]. This workflow has been previously used to investigate the transcriptome of adenoviruses, including HAdV-C5, ChAdOx1, an adenovirus-based vaccine against HIV and a fowl adenovirus identifying conservation of key elements of the adenovirus genome [22–25].

We sought to characterise the capsid structure and viral transcriptomic profile of HAdV-D10. We present the first high-resolution map of HAdV-D10 and a map of the fiber protein revealing an uncharacterized fiber shaft ‘umbrella’ motif. We observed transcription levels which varied from those observed in both HAdV-C5 and ChAdOx1 vectors, including production of expected pVII transcripts and additional transcripts equivalent to mature protein VII. Our study highlights the largely conserved structure of human adenovirus capsids across species while providing highly detailed transcriptomic data, adding to the portfolio of information surrounding HAdV-D10 to aid its development into a therapeutic vector.

## Results

### Structure of HAdV-D10 virions

**Fig 1:**
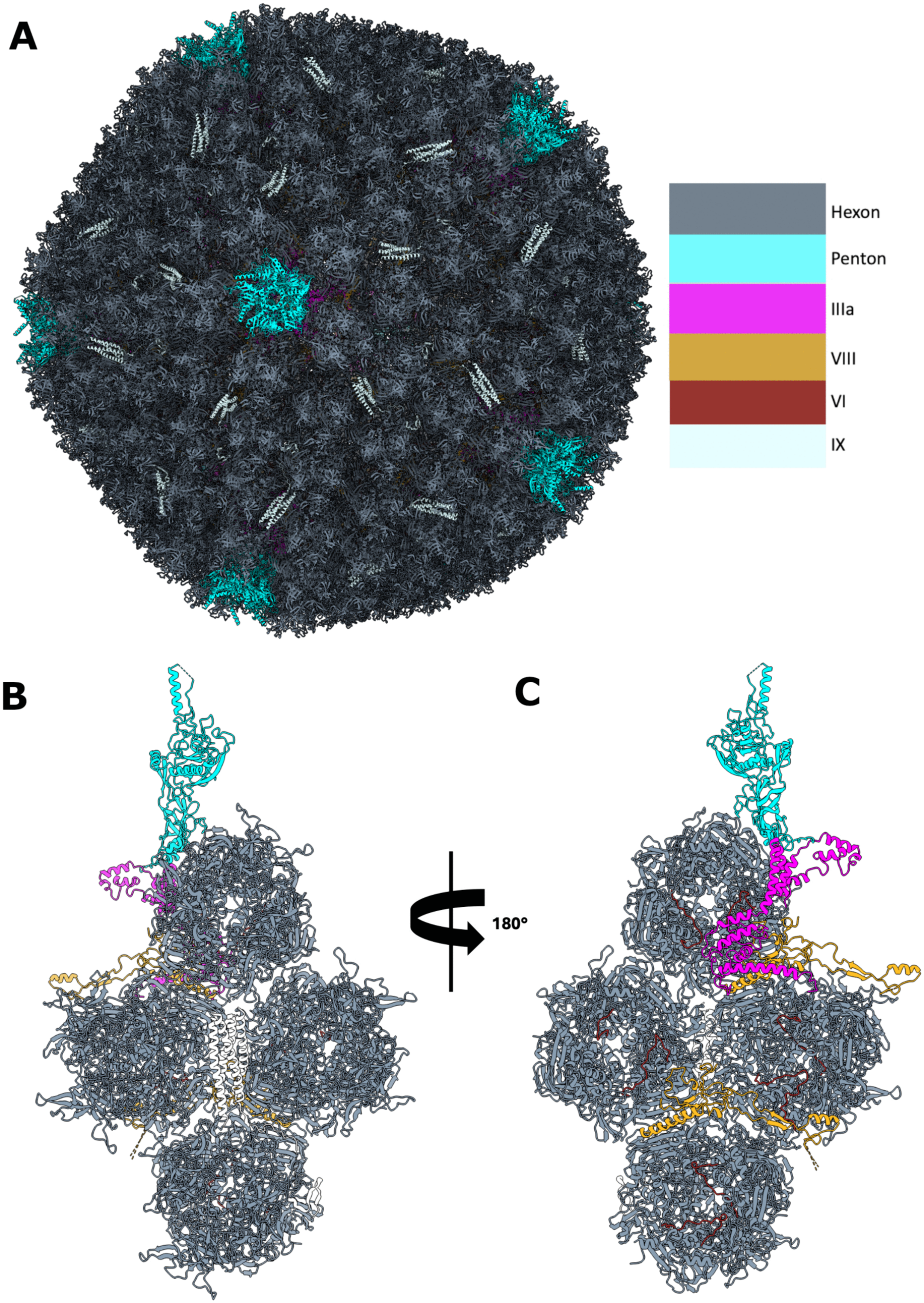
Structure Of HAdV-D10. Molecular model of biological assembly of HAdV-D10 is shown in cartoon style in panel A with a colour key highlighting protein components. Panels B and C show rotated views of the modelled asymmetric unit that repeats to form the biological assembly. Shown are exterior (B), and interior (C) capsid views respectively displaying modelled major proteins hexon and penton and minor proteins IIIa, VIII, VI and IX.

Single particle analysis using icosahedral symmetry led to the calculation of a capsid reconstruction at 3.3 Å resolution using a total of 5,524 capsid particles. The structure of the HAdV-D10 capsid is largely similar to previously published mastadenovirus structures with global pseudo-T = 25 icosahedral. The sharply resolved map supported the assembly of models for hexon, penton, and proteins IX, VIII, IIIa and VI. To resolve the symmetry mismatched fiber and penton base, focused classification was used to first identify intact fiber shaft particles followed by focused refinement to resolve the fiber shaft at a global resolution of 4.6 Å resolution with the fiber shaft itself at around 10 Å resolution by local resolutions estimates. Fig 1A presents the icosahedral assembly of the HAdV-D10 capsid. Fig 1B presents an exterior view of the repeating asymmetric unit of the capsid and Fig 1C presents an interior view of the asymmetric unit. The cryo-EM data used for building the atomic model are shown in S1 Fig. The modelling statistics for the asymmetric unit of the HAdV-D10 capsid are listed in S1 Table. The structure of highlighted minor capsid proteins are presented in S2 Fig.

### Hexon

The full length, 949 amino acid, atomic model could be built for hexon protein. The overall architecture of hexon protein closely matches HAdV-C5 and HAdV-D26 with root mean square deviations (RSMD) of 1.158 Å and 2.448 Å respectively (S3 Fig).

### Penton base

The penton base (coloured in cyan in Fig 1A-1C) is highly conserved compared to HAdV-C5 (RMSD 0.762) and HAdV-D26 (RMSD 0.492) (S3 Fig), and similarly is well resolved except for residues 299–324 which correspond to the loop containing the integrin binding RGD motif (Figs 1B, 1C and S3).

### Minor proteins

The protein IX structure reported here supports protein IX is mostly conserved across human species C and D adenovirus with some dissimilarity in contrast to previously published HAdV-C5 and HAdV-D26. Data for HAdV-D10 is far less complete than HAdV-C5 and HAdV-D26 where a more complete protein IX network was modelled in HAdV-C5 the most complete chain models residues 7–57 and 67–134. HAdV-D26 is the most complete with almost the entire molecule modelled residues 2–134. The HAdV-D10 cryo-EM data is sufficient to model the coiled-coiled domain which is conserved in HAdV-C5 and HAdV-D26 of protein IX and for one chain the linker region 82–96 is modelled, a loop region which connects the coiled-coiled domain to a short disordered domain 56–76 that latches the side of hexon. Only one of the hexon-latching domains could be modelled for HAdV-D10 chain S. This is a notable difference to HAdV-C5 where protein IX has three chains modelled in density for hexon-latching domains. This is likely due to the HAdV-C5 dataset being much larger ~45,000 particles compared to ~5,000 which will greatly aide averaging improving map quality. In further contrast to HAdV-C5, protein IX chain S in HAdV-D10 (corresponds to Q in HAdV-C5 structure) latches hexon 3 in the asymmetric unit whereas the same protein IX chain in HAdV-C5 latches hexon 2 (S2A Fig). The protein IIIa model is supported well by the cryo-EM data with residues 2–283 modelled (S2C Fig). Protein IIIa is conserved compared to HAdV-C5 and HAdV-D26 with RMSD of 2.068 Å and 0.979 Å respectively. Unlike the reported HAdV-D26 structure there is no data to support a model for the protein IIIa appendage domain (APD domain) in the HAdV-D10 structure reported here [26] (S2C Fig). Eight copies of VI were modelled internal to the hexon with two copies of VI modelled in all four hexons of the asymmetric unit. No other minor capsid protein could be modelled.

### Fiber shaft

**Fig 2:**
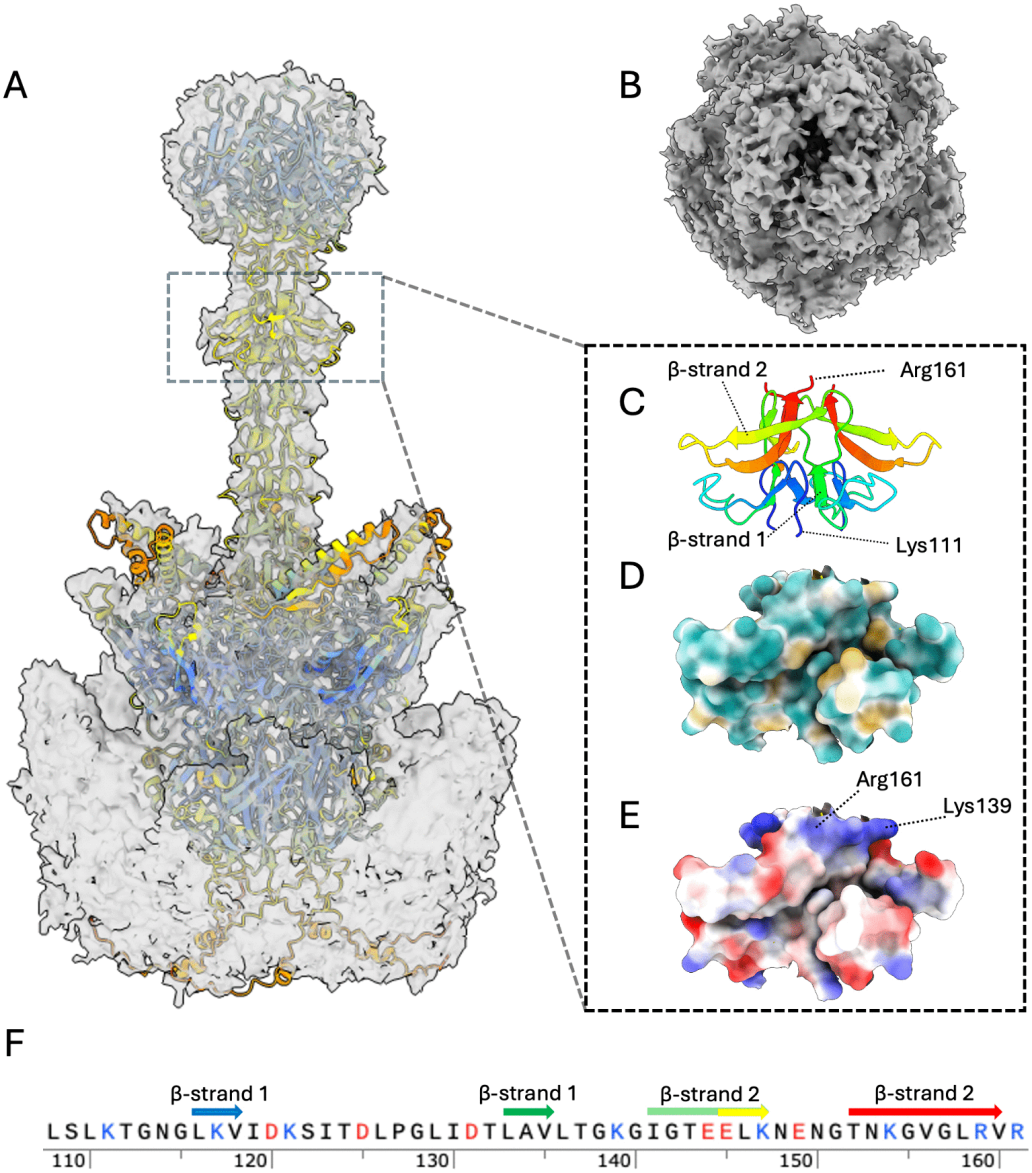
Structure Of HAdV-D10 Fiber. Panel A presents a focused refinement cryo-EM map of major protein fiber at 4.6 Å resolution with an AlphaFold3 predicted model of the trimeric fiber shaft and pentameric penton fitted into the map. The AlphaFold model is coloured by standard pLDDT confidence scores colours dark blue >90, light blue 90 - 70, yellow 70 - 50 and orange <50. Fiber ‘umbrella’ motif is highlighted with a dotted box. Panel B a top down view of the fiber protein illustrating the fiber knob. Panel C shows view of fiber motif with rainbow colour scheme to illustrate fold. N- termini to C-termini residues are coloured blue, cyan, green, yellow then red. Hydrophobic colouring and surface representation of fiber motif are presented in panel D. Coulombic colouring and surface representation of fiber motif are shown in panel E illustrating electrostatic properties of residues. Panel F sequence of umbrella motif residues 111-161 with charged residues coloured and secondary structure elements labelled and coloured by N- to C- termini rainbow colour scheme as in panel C.

We used focused classification and subparticle refinement to determine the structure of the flexible fiber shaft (Figs 2, S4 and S5). The comparatively short length of HAdV-D10 shaft at 150 Å likely reduces flexibility aiding classification. Thus, we were able to resolve the penton-fiber structure at a global resolution of 4.6 Å, local resolution estimates the fiber at 10 Å (S4 Panel A). The length of the HAdV-D10 fiber is comparable to HAdV-D26 for which the fiber shaft structure was previously classified using a local reconstruction method [27]. The quality of our map allowed validation of an AlphaFold3 prediction for the penton-fiber structure, fitted as a rigid body in our experimental data. Although the local resolution of the fiber shaft itself is around 10 Å the map has a clear protrusion two thirds of the way up the fiber shaft not identified in HAdV-D26 [27]. The protrusion of density in the HAdV-D10 fiber shaft map matches well to the AlphaFold model of fiber shaft which has the same protrusion formed of residues 111–161 and is confidently predicted with pLDDT scores of between 65–95 when the fiber is predicted alone and 65–78 when the fiber and penton are predicted as a complex (S2 Table). In the Alphafold prediction this region folds to form two β-strands. The shorter β-strand 1, consists of residues 133–135 and 116–119 that forms the final repeat of the β-strand structure that forms the fiber shaft structure. The second longer β-strand 2, flaring away from main fiber structure with the β-strand consisting of residues 153–162 and 141–147 presenting a short solvent exposed loop 148–154 creating the notable protrusion identified in the fiber shaft EM structure (Fig 2C and 2F). The resulting motif has intriguing electrostatic properties with a positive charge patch present at the apex of the motif formed of residues Lys139 and Arg161 and the solvent exposed loop containing a negatively charged Glu149 (Fig 2E and 2F). Given the resulting shape the motif forms we have termed residues 111–161 the fiber shaft ‘umbrella motif’.

**Fig 3:**
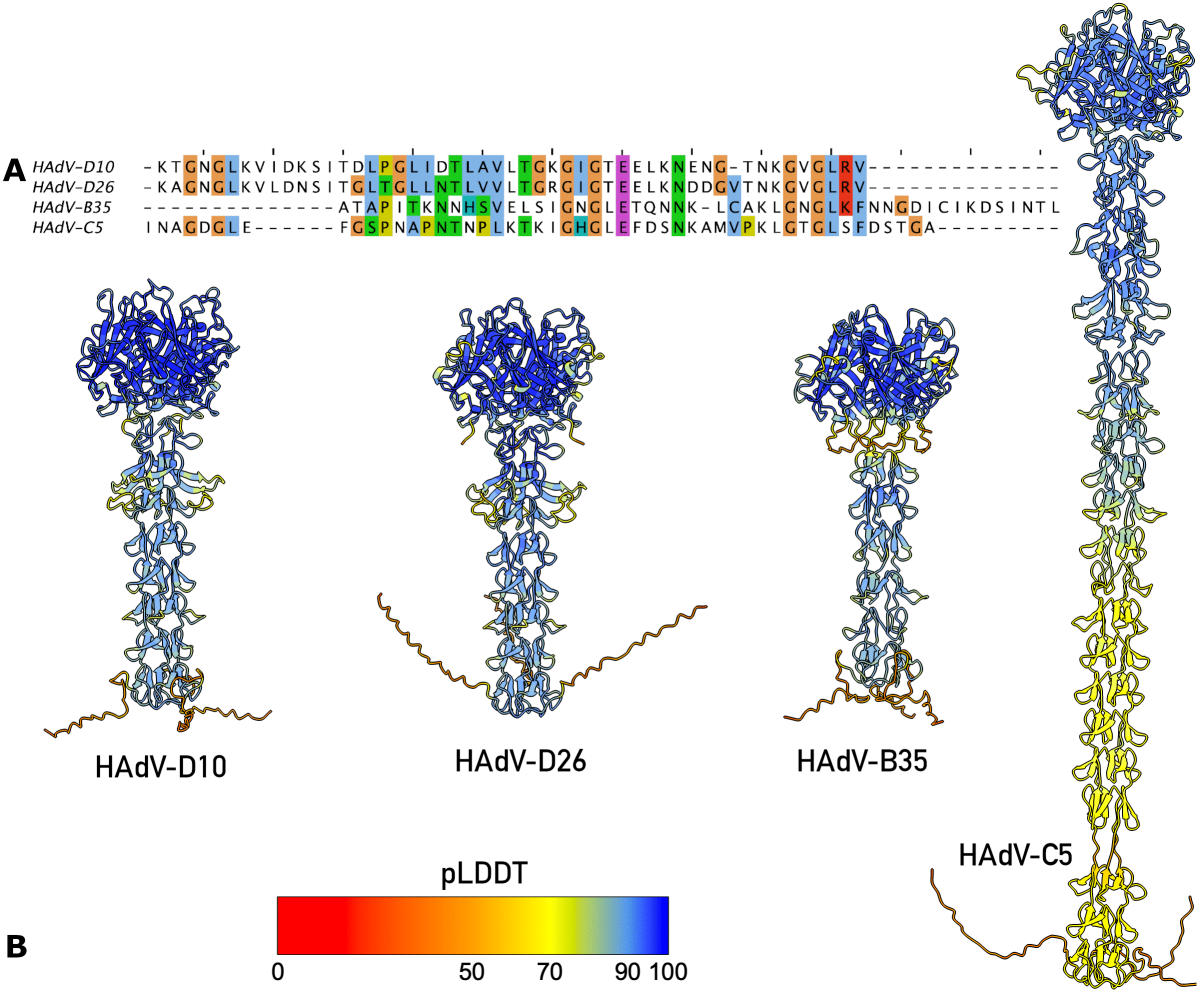
Comparison of HAdV Fiber Shaft Structures. Panel A presents sequence analysis of fiber shafts after structural alignment to umbrella motif residues 111-161 of HAdV-D10. Panel B presents a comparison of AlphaFold3 modelled structures of HAdV fiber shafts coloured by standard pLDDT confidence scores, colour key displayed. Comparison of shaft structures shows species D serotypes HAdV-D10 and HAdV-D26 have similar sequence identity and the umbrella motif predicted.

The experimental validation of this motif suggests AlphaFold3 predicted structures for other species D fiber are likely accurate. The motif is also predicted for HAdV-D26 with HAdV-D10 sharing sequence similarity of 76% when aligned structurally (Fig 3A). A similar motif is not predicted for HAdV-B35 despite the shaft being a similar length. The motif is also not predicted for HAdV-C5 (Fig 3B).

### Transcript maps of HAdV-D10 show production of key adenovirus genes

**Fig 4:**
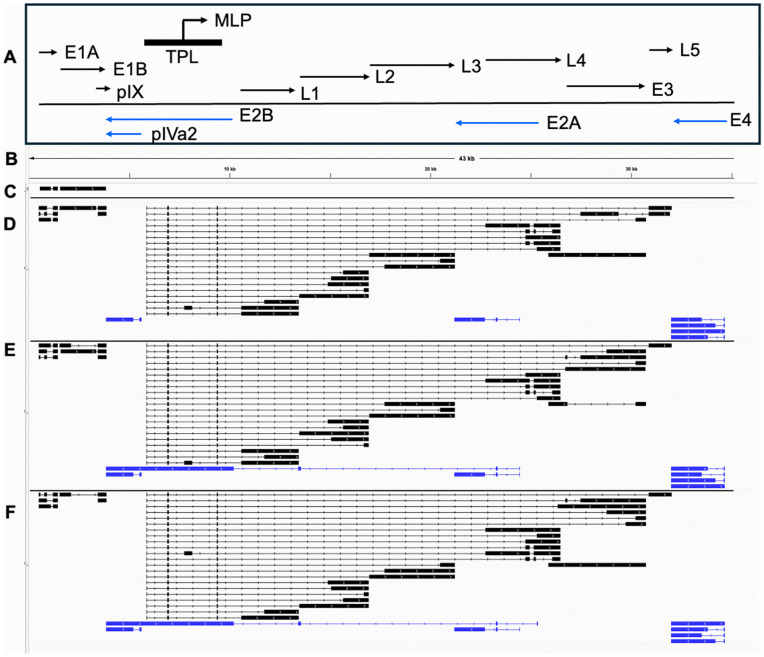
Transcription map of HAdV-D10 across multiple timepoints. Images were made in IGV [28–30]. Panel A presents an overview of the classical HAdV-C5 transcriptome map with key genes highlighted. Panel B presents the HAdV-D10 genome which the following panels are aligned to. Transcript maps with the most abundant transcript for each of the known HAdV-D10 proteins are presented for 6 hours post infection (panel C), 24 hours post infection (panel D), 48 hours post infection (panel E) and 72 hours post infection (panel F). All transcripts are grouped according to strand, with forward strand coded transcripts shown in black and reverse strand coded transcripts shown in blue. Exons are presented as rectangles, and introns as lines, with arrows highlighting the direction of the strand.

The ORF-centric analysis pipeline for the dRNA-seq data generates a transcript map showing the structure of the most abundant transcripts for each ORF present on the HAdV-D10 genome. Fig 4A presents a general overview of the classical HAdV transcriptome map with key genes highlighted and labelled with direction of transcription, aligned approximately to HAdV-D10’s genome (Fig 4B). Fig 4 presents the most abundant transcripts for each ORF for each of the 6 (Fig 4C), 24 (Fig 4D), 48 (Fig 4E) and 72 (Fig 4F) hours post infection (h.p.i.) timepoints investigated for HAdV-D10 infected cells displayed in Integrative Genomics Viewer (IGV) [28–30]. The maps for each of our timepoints correlates with classical adenovirus transcriptomes previously investigated, as the locations of the identified genes are the same as observed for HAdV-C5 [22]. Only the E1 region was detected at 6 h.p.i., therefore this dataset was not included in further analysis.

### Change in expression of HAdV-D10 genes over time follows the expected profile

**Fig 5:**
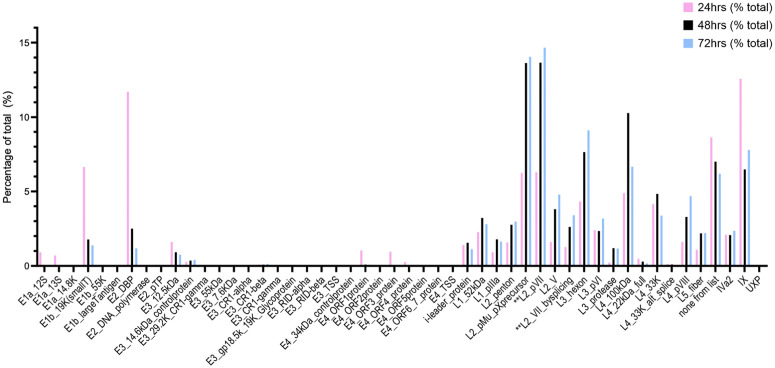
Change in transcription of known HAdV-D10 genes over 24-, 48- and 72-hours post infection. The percentage of total transcripts for each known HAdV-D10 gene are shown across the three timepoints, with values for 24 h.p.i. shown in pink, 48 h.p.i. shown in black and 72 h.p.i. shown in blue. Transcripts encoding L2_preVII or L2_VII_bysplicing have been highlighted with asterisks.

Adenovirus genes are categorized by temporal expression in the viral infection pathway, with early, intermediate and late genes being expressed sequentially [22]. Fig 5 highlights the change in percentage of total transcripts produced per gene across the three timepoints at which RNA was sequenced. Higher percentages of early genes (E1, E2, E3 and E4 genes) are seen at 24 h.p.i. (pink bars), with increasing levels of late gene transcripts observed at 48 h.p.i. (black bars) and 72 h.p.i. (blue bars). At the end of the graph are the intermediate gene transcripts, with similar expression across the three timepoints.

S3 Table presents the percentage of transcripts which code for any given ORF. A transcript is defined as coding for an ORF when the 5’ most start codon codes the initiating methionine of that ORF. This does not always add up to 100% because not all transcripts code for a known ORF and in some cases a protein’s expression may result from initiation of a downstream start codon (perhaps because of poor Kozac consensus).

At the 24 h.p.i. timepoint, the gene with the highest percentage of transcripts being expressed is the intermediate gene encoding protein IX with 12.576% of total transcripts. This is closely followed by the E2 DNA binding protein with 11.703% of total transcripts at 24 h.p.i.. At the 48 and 72 h.p.i. timepoints, the L2 genes pVII and pMu are most abundant. pVII accounts for 13.651% of all transcripts at 48 h.p.i., and 14.663% of all transcripts at 72 h.p.i. making it the most produced transcript by HAdV-D10 at later timepoints. pMu is also highly expressed, with 13.630% of transcripts at 48 h.p.i. and 14.051% of total transcripts at 72 h.p.i.

Two E2B genes are so rarely transcribed, they account for less than 1% of total transcripts each per timepoint [22]. The DNA polymerase was detected at each timepoint, but at such low levels that it accounts for less than 0.500% of all transcripts across the timepoints, whereas pTP has slightly increasing percentages, from less than 0.500% at 24 h.p.i., to 0.005% and 0.004% at 48 hours and 72 h.p.i..

### HAdV-D10 produces two distinct transcripts coding for precursor and mature pVII

**Fig 6:**
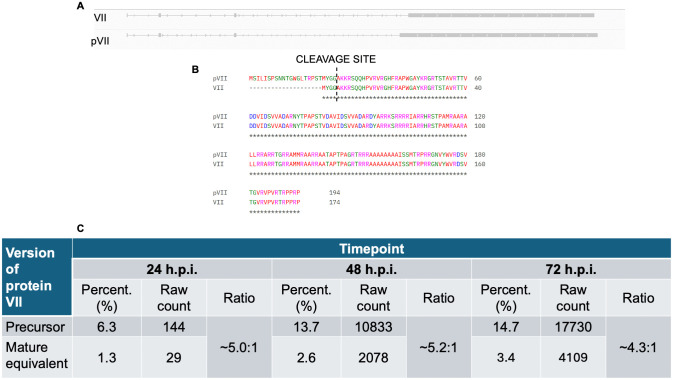
Production of preVII and pVII transcripts by HAdV-D10. Panel A illustrates HAdV-D10’s preVII and pVII transcripts observed in IGV [28–30]. The transcript equivalent to mature pVII is shown first, with preVII underneath. Panel B presents the sequences of the two different transcripts, aligned by Clustal Omega [31]. The cleavage site of the adenovirus protease is displayed for both of the sequences. Panel C presents the ratio of precursor to mature equivalent pVII transcripts at 24, 48 and 72 h.p.i.

Closer analysis of data presented in Fig 5 and S3 Table revealed two transcript groups coding for protein VII were being produced, highlighted by two asterisks (Fig 5 and S3 Table). The expected pVII transcript was produced at 24, 48 and 72 h.p.i. (Fig 5), exceeding 10% of total transcripts produced in the 48 and 72 h.p.i. data sets. In contrast, a transcript coding for a protein equivalent to mature VII was produced at less than 5% of all transcripts across all three timepoints. Fig 6 highlights the differences between these two transcript groups in the 24 h.p.i. timepoint, with the transcript coding for a protein equivalent to mature protein VII being generated due to utilization of a splice site located in the precursor region of pVII, therefore the precursor region is not present in the equivalent to mature VII transcripts (Fig 6A).

The transcripts for pVII and the equivalent to mature VII both contain the tripartite leaders, and begin at the MLP alongside other late expressed genes (Fig 4). Their sequences were aligned using Clustal Omega [31] and the output is shown in Fig 6B. The pVII transcript has an extension at the 5’ end, and the cleavage site for the adenovirus protease according to previous work [32] is highlighted after the first initiating methionine of the transcript coding for the equivalent to mature protein VII, and after the second methionine for the pVII transcript.

At the earlier timepoints, there is a higher ratio of precursor to equivalent to mature transcripts (Fig 6C), whereas at 72 h.p.i., pVII has a much higher raw count, at 17,730 transcripts accounting for 14.663% of all transcripts (Fig 6C). Although the transcript equivalent to mature protein VII accounts for a smaller percentage of total transcripts at 3.4%, it is the 9^th^ most abundant transcript for this timepoint, exceeding the percentage for major protein coding transcripts such as the L5 fiber gene. This hints at a separate role for protein VII in the HAdV-D10 lifecycle that we are currently unaware of. The two transcripts are being produced at a ratio of approximately 4.3 pVII transcripts to 1 transcript equivalent to the mature protein VII.

### Hypothetical proteins from NCBI gene were identified in HAdV-D10 sequencing outputs

Fig 5 highlights a large percentage of transcripts categorized as ‘none from list’. These transcripts account for 8.600% of all transcripts at 24 h.p.i., 7.000% of all transcripts at 48 h.p.i. and 6.200% of all transcripts at 72 h.p.i.. These are large percentages compared to other transcripts being produced, with this category being the 3^rd^ most abundant transcript group at 24 h.p.i.

Using the National Centre for Biotechnology Information’s (NCBI) Blast tool [33], it was determined that most of these transcripts were truncated HAdV-D10 transcripts. This was not true of all the unidentified transcripts, and Table 1 presents three transcripts which were not identified as HAdV-D10 transcripts.

**Table 1:** Hypothetical transcripts produced by HAdV-D10 infected cells identified by NCBI BLAST [33]. Three hypothetical transcripts previously reported in NCBI Gene were identified via BLAST searching unknown transcripts. Their relative abundance has been combined for all three timepoints (labelled Transcript Abundance) and the Accession IDs associated with the hypothetical transcripts have been reported.

Transcript Abundance	Start	Splicing Events	End	Sequence	Accession ID
227 copies	16,390	NONE	16,955	MGRRQPADAQLRVASFHHPDAGLPRHPVLRQPPAPSQQTPPPHRHPPPSGPRPRAPRDHAPGPLARSAHRALPPQHPLIRVL	UKB92904.1 or UKB93056.1
86 copies	5,858	5,898 – 6,920 – 6,991 – 7,761 – 8,167 – 16,697	16,955	MRADWEELDFLPPVGRVAVDVMKVEIPPANRALVLMLVKASAVLAALHGLYLIHEIHSASLEEELQEWRPWLVAFMFACVGLTLGLLEDGEADEPAREPGPDLGAAGAESEDEGAQLGAVHGVAEIQRDGSHLPPAHPRPELPRKIPPQERHGRQRPEPPPAAGHAQAPEWRLSARAHPHNRRGHRHDPGHSFRCAAGVAAPLMCE	UYO78390.1
52 copies	5,858	5,898 – 6,920 – 6,991 – 7,761 – 8,167 – 16,697	16,955	MRADWEELDFLPPVGRVAVDVMKVEIPPANRALVLMLVKASAVLAALHGLYLIHEIHSASLEEELQEWRPWLVAFMFACVGLTLGLLEDGEADEPAREPGPDLGAAGAESEDEGAQLGAVHGVAEIQVRGQGSEVDLVEAGEGVLEMQMVLDLHG	AGT76484.1 or UYO78390.1

The start, end and splicing events are noted for each transcript, alongside their sequence and abundance across all three timepoints. After NCBI Blast searches, these were identified as hypothetical HAdV transcripts. Each Accession ID is noted in Table 1. These sequences are hypothetical, noted to have been predicted to be produced by human adenovirus genomes. The first predicted transcript listed in Table 1 is not spliced, and is initiated and terminated inside of the L2 coding region. The second and third transcripts are heavily spliced, utilising canonical GU:AG splice sites, bolstering the likelihood that they are authentic transcripts. They would be initiated at the MLP at base 5,858, and terminate in the L2 region.

## Discussion

HAdV-D10 is of interest for therapeutic applications including oncolytic virotherapies and viral vaccine vectors [8]. Due to the infrequent nature of infections [6], we sought to extend the knowledge base surrounding this virus to aid further engineering and investigate its safety profile. By engineering the A20 peptide from Foot and Mouth Disease Virus (FAMDV) which binds the αVβ6 integrin [34] into the HAdV-D10 fiber knob protein, we were previously able to target HAdV-D10 to cells overexpressing the integrin, including several epithelial cancers [8]. Previous literature has centered around HAdV-D10 pathology [4–6], therefore exploring the viral structure and transcriptomic profile enables further insights into the potential of HAdV-D10 as a therapeutic vector.

Prior structural studies have been important for identifying host interactions with viral capsid proteins [14,35]. In this study, we produced a 3.3 Å resolution structure of the HAdV-D10 capsid (Figs 1A and S1). This largely follows the expected structure of major and minor proteins for a human adenovirus species C and D capsid, previously determined by cryo-EM [10,11] as observed in Figs 1, S2 and S3.

HAdV capsids are 80–100 nm in diameter, excluding the fiber, making cryo-EM the appropriate technique for whole capsid investigation [36]. The fiber projects away from the core capsid, anchored by the penton base as observed in Fig 2A. There are 12 fibers and pentons per capsid, found at each vertex [11]. The fiber protein shaft is made up of variable numbers of a repeating motif, therefore the length differs across species [37]. HAdV-D26 was observed to have a shorter, more rigid fiber shaft than HAdV-C5, making it easier to observe by cryo-EM; however the reported map for HAdV-D26 is not of sufficient quality to validate the predicted ‘umbrella motif’ reported here [11]. The symmetry mismatch between the pentameric penton and trimeric fiber shaft makes resolving this anchoring interaction challenging [38]. The 10 Å resolution and quality of the fiber shaft map reported here does not resolve the fiber shaft tail insertions however has allowed reasonable fitting of the confidently predicted AlphaFold3 fiber shaft model identifying the novel umbrella motif formed of residues 111–161 (Fig 2A and S1 Table). The motif may act as a secondary mechanism of virus-host receptor engagement with residues Lys139 and Arg161 forming an electropositive patch (Fig 2D - 2E). The motif presents typical features of a binding region, with a core network of hydrogen bonded residues forming a stable structure, in this case two β-strands, that present an exposed loop to engage receptors through hydrogen bonding, electrostatic or hydrophobic interaction. For example, penton protein forms a stable hydrogen bonded α-helix, residues 282–298 reported here, that extends and projects a flexible loop into the solvent exposed space. The flexible loop contains an RGD motif that engages with host cellular integrins and corresponds to residues 299–324 in HAdV-D10 [16].

As further evidence serotypes should be well characterised prior to clinical translation, the detailed transcriptomic profile of HAdV-D10, while similar in gene locations, deviates from the profile of HAdV-C5 [22]. Although the transcriptome maps (Fig 5C - 5E) align with that produced by HAdV-C5 using the same ORF-centric analysis pipeline [22] and an independently produced adenovirus 2 (HAdV-C2) dataset [39], diving into the individual transcripts and their varied production over the three timepoints revealed unexpected changes (Fig 6). While the conservation of the transcriptome map and ORFs identified in HAdV-C5 is positive from a vector development perspective, as HAdV-C5 has already been engineered as an oncolytic virus [40], it is important to investigate the differences as these highlight important safety considerations when developing different adenoviruses as vectors. This is highlighted by transcriptomic investigations into the ChAdOx-1 nCoV19 vaccine [23]. Small changes from viral engineering could alter splice sites or promoter usage, introducing unintended changes. Long read sequencing confers an advantage for investigating adenovirus transcriptomics as we can investigate the high level of splicing and usage of multiple ORFs seen across adenovirus genomes, including HAdV-D10 [41,42].

The differences were especially true in the case of protein VII. It is normally made in a precursor form (pVII) and processed to mature protein VII by the viral protease [43,44]. We noted some transcripts equivalent to mature protein VII were produced by HAdV-D10 infected cells in addition to transcripts expected for pVII, at a ratio in the 72 h.p.i timepoint of approximately 4.3 precursor coding transcripts to 1 mature protein VII coding transcript (Fig 6C). Both pVII and mature protein VII interact with DNA in the nucleoprotein core [44,45]. HAdV-D10 infected cells are producing a far higher percentage of total reads for pVII and equivalent to mature protein VII, compared with major structural protein coding transcripts such as the genes encoding the hexon and fiber (Figs 5 and 6), suggesting that, as in HAdV-C5, the stoichiometry of gene transcripts to genes does not reflect the number of protein copies in a viral capsid [22].

We revisited the HAdV-C5 dataset and a single transcript coding for mature protein VII was found [22], which may suggest an undetermined role or increased need for protein VII by HAdV-D10 to explain the increased number of transcripts produced. Protein VII has been described as a histone-like protein and is thought to have roles including evading the host immune system, and modulating the host’s DNA damage response during HAdV infection [46,47]. Our data (Fig 5) also highlighted a higher percentage of total transcripts for the gene encoding pMu (almost 15%) compared to HAdV-C5 (approximately 8%, Fig 2 of Donovan-Blanfield et al. [22] where pMu is referred to as pX), which has been suggested to modulate splice site selection during adenovirus infection [22,48]. This protein is also involved in binding DNA and localises in the nucleoprotein core, as with pVII/protein VII [45,48] strengthening the position that HAdV-D10 may express DNA binding genes at higher levels relative to HAdV-C5. DNA condensation is extreme in adenoviral capsids. Large amounts of DNA must be packed into the 80–100nm icosahedral capsid and protein Mu, VII and V have been reported to work similarly to eukaryotic histone proteins to condense adenovirus DNA, interacting with each other to form adenosomes which can be observed via cryo-electron tomography (cryo-ET) [21,43]. An important note when interpreting this data, is that Kyse-30 cells are not retinal and therefore do not originate from the natural target tissue of HAdV-D10 [5]. As a result, the transcriptional profile of HAdV-D10 may vary in tropism-relevant primary cells or cell lines.

Why HAdV-D10 might require relatively higher levels of DNA binding proteins compared to HAdV-C5 requires more exploration, to elucidate the interplay of the different proteins produced by HAdV-D10 in host cells. Proteomic investigations may aid this, to confirm if the high number of transcripts correlates with levels of protein production, or if the stoichiometry is unequal as seen in HAdV-C5 [22]. Proteomic analysis would also help confirm production of the hypothetical transcripts identified in Table 1 are protein encoding, as performed previously alongside dRNA-seq data [41].

In conclusion, we have combined the first whole capsid structure of the HAdV-D10 capsid at 3.3 Å (Figs 1 and S1) with the first transcriptomic investigations (Fig 4) of the same virus, revealing unique features and insights into its biology. We have also provided a 10 Å map of the fiber shaft revealing a previously uncharacterised motif (Fig 2). While mostly sharing structural characteristics to HAdV-C5 [10], there are key variations in the transcriptomic data including the presence of hypothetical transcripts and unexpected transcripts equivalent to mature protein VII (Fig 6 and Table 1). While these differences require further proteomic investigation, HAdV-D10 remains a promising potential vector for therapeutic application. The combined structural and biological information presented in this work can be used to improve safety and efficacy of future HAdV-D10-based vectors and guide vector engineering strategies.

## Materials and methods

### Virus production and purification

All viruses were produced as previously described [49]. Briefly, viruses were produced in E1A complementing cell lines and were purified by multiple rounds of caesium chloride (CsCl) gradient ultracentrifugation before dialysis against a buffer made up of 10% glycerol, 135mM NaCl, 10mM Tris-HCl (pH 7.8) and 1mM MgCl_2_(H_2_O)_6_. A viral titre was determined by microBCA assay (Pierce microBCA protein assay kit, ThermoFisher Scientific, Waltham, USA, Catalogue number: 23235) and by immunostaining assay to determine the functional titre. To convert the microBCA result to a viral titre, the formula 1µg protein = 4 x 10^9^ viral particles/mL was used [50,51]. The wild-type HAdV-D10 virus used for transcriptomic experiments had a measured functional titre of 3.26x10^10^ PFU/mL while the replication deficient HAdV-D10 virus used for cryo-EM had a viral titre of 3.02x10^12^ VP/ml by microBCA assay.

### Cryo-EM grid preparation

Grids of purified HAdV-D10 were prepared using Quantifoil R2/2 Cu 300 mesh grids, covered in-house with a thin layer of carbon produced using a Quorum Q150. Grids were glow discharged using a Emitech K100K for 30 seconds at 15 mA in air. Plunge freezing was performed using a Vitrobot Mark IV into liquid ethane. The Vitrobot chamber was set to 95% humidity and 4.5°C. 6 ul of sample was applied and, after a 60 second wait time, blotted for 6 seconds with a blot force of -6.

### Cryo-EM data collection

Data collection for the high-resolution reconstruction was performed at Diamond Light Source Electron Bio-Imaging Centre (eBIC) using a Thermofisher Titan Krios equipped with a Gatan K3 detector operated in super resolution mode. Exposures with a total dose of 40 e/Å2 were recorded over 40 frames, and a nominal defocus range of -2.2 µm to -0.2 µm. In total 22,490 micrographs were collected. The recorded images were binned by two, resulting in a calibrated pixel size of 0.829 Å/pixel at the image level.

### Single particle data analysis of HAdV-D10 Capsid

Single particle analysis was performed in RELION 3.1 [52] using a high-performance computing cluster (HPC) with GPU acceleration from NVIDIA P100’s. Briefly, motion correction was performed using MotionCor2 [53]. Contrast transfer function was estimated using GCTF [54], with poorly estimated micrographs, based on the fit of the real CTF to the theoretical CTF, removed from further processing by visual inspection of the Thon rings. 714 micrographs were removed at this stage. 100 particles were manually picked and used as a 2D class templates for RELION’s “Autopicker” resulting in a dataset set of 11,344 particles. Particle false positives were removed by multiple iterative rounds of 2D classification. An ab-initio model was generated from the data using the RELION’s stochastic gradient descent algorithm without the imposition of symmetry. The Ab-initio model was subsequently aligned to I2 symmetry using “relion_align_symmetry.” The I2 aligned Ab-initio reference map was used as a reference to refine particles at 5x, 4x and subsequently 3x Fourier cropping factors, re-extracting the particles after each refinement reached the Nyquist limit of the Fourier cropped pixel size. Particles were then re-extracted in a box of 1440 pixels (~1194 Å) Fourier cropped by a factor of two to 720 pixels resulting in a sampling rate of 1.658 Å/pixel. This was the maximum box size allowed within memory constraints of the HPC. After multiple rounds of iterative CTF and 3D refinement with per-particle defocus, astigmatism, beamtilt and trefoil the resulting map has a resolution of 3.8 Å with 5,524 particles included in the reconstruction. After Ewald sphere correction, the resolution estimate for the capsid by gold standard Fourier shell correlation (FSC) calculations is 3.3 Å.

### Model building

“Phenix_sharpen” was used to sharpen the unfiltered half maps [55]. SwissModel [56] and the Alphafold3 server [57] were used to generate homology models for the capsid proteins. Each homology model was first fitted into the map using ChimeraX’s “fit in map” function as rigid bodies [58]. The models were then independently adjusted, particularly for C-⍺ backbone fit into data, using COOT [59]. Once all models with experimental data were independently built, ISOLDE [60] was used to refine the whole asymmetric unit, first allowing the asymmetric unit model to relax into the map by running an all-chain molecular dynamics AMBER forcefield simulation with a temperature of 20 K. Subsequently the temperature was lowered from 20 K to 1 K and individual residues were inspected to improve side chain fit to data, rotamer angles and Cα Phi and Psi angles [60]. Finally, one round of phenix real space refinement was performed to generate the .cif file. The biological icosahedral assembly, shown in Fig 1A, was produced by symmetry-related copies using Chimera’s “Sym” command.

### Focused classification and refinement of fiber shaft

The fiber shaft map was determined using targeted masking, 3D classification and refinement without the application of symmetry (C1) within RELION 3.1. First, the refined capsid particles from the 3.3 Å map were re-extracted and Fourier cropped by a factor of 5 from the previously used box size of 1440 pixels (1194 Å) resulting in a sampling rate of 4.145 Å/pixel. “Refine3D” was then used to output a particle.star file required for “relion_symmetry_expand” a RELION script that expands the particle set by the applied symmetry group, in this case I2 symmetry. A cylindrical mask that covered the diameter of the penton and spanned the expected length of the HAdV-D10 fiber shaft was made using MATLAB and DYNAMO [61]. The mask was placed over the penton and fiber shaft resampling onto the grid of the capsid map using ChimeraX. RELION “MaskCreate” was then used to apply a soft edge to the cylindrical mask. “3D Classification” was then performed using the expanded symmetry particles, Fourier cropped capsid reference map and cylindrical mask without performing image alignment. The resulting classes were assessed for intact fiber shaft particles, which were subsequently pooled, and re-extracted in a 1440 pixel box Fourier cropped to 720 pixels resulting in a sampling rate of 1.658 Å/pixel. Using the intact fiber shaft particle coordinates “Particle Subtraction” in RELION was performed using the same cylindrical mask in a new smaller box size of 200 Å subtracting the capsid signal away from the fiber shaft signal. Using the new signal subtracted particle substack of fiber shaft particles, 3D classification was performed with image alignment, using a regularisation parameter of 4, performing local angular searches from 5 degrees, for 25 iterations and filtering particles into 5 classes. 3 classes had full length fiber shaft particles were selected and pooled for refinement. The subsequent converged refinement was sharpened by b-factor value of 139.05 using RELION’s “PostProcess” resulting in the final map shown in Figs 2A, S4 and S5.

### AlphaFold structure prediction

All AlphaFold predictions reported here were generated using the AlphaFold3 server [57]. For the Fiber-Penton model, five copies of the penton and three copies of the fiber sequence were submitted to the AlphaFold3 server as two separate protein entities. The model was then manually placed into the cryo-EM map and then fitted using ChimeraX’s “fit in map” command.

### RNA extraction, mRNA enrichment and sequencing

Kyse-30 cells were grown to a minimum of 80% confluency in T75 flasks. Cells were infected with wild-type HAdV-D10 at an MOI (multiplicity of infection) of 10 determined by immunostaining assay.

RNA was extracted 24 h.p.i. using previously described methods utilising TRI reagent (ThermoFisher Scientific, Waltham, USA, Catalogue number: AM9738) and chloroform [22]. The resulting pellet following isopropanol precipitation was washed three times with ethanol to increase purity.

The DynaBeads mRNA Purification kit (ThermoFisher Scientific, Waltham, USA, Catalogue number 61006) was used to prepare total RNA extracted for polyA-tail enrichment according to manufacturers’ instructions. Resulting mRNA was then used for sequencing. The dRNA-seq kit from Oxford Nanopore Technologies (Oxford, UK, Catalogue number: SQK-RNA004) was used according to manufacturers’ instructions to prepared dNRA-seq libraries and perform sequencing using a MinION Mk1C (Oxford Nanopore Technologies, Oxford, UK, Catalogue number M1CBASICSP) and RNA flow cells (Oxford Nanopore Technologies, Oxford, UK, Catalogue number: FLO-MIN004RA). Data was acquired over 72 hours per sample achieving the following number of reads for each run: 876.140 thousand for 24 h.p.i., 2.290 million for 48 h.p.i. and 3.040 million for 72 h.p.i..

### Transcriptomics data analysis

An ORF (open reading frame)-centric pipeline was applied to the data output from the MinION Mk1C as previously described [22]. Reads were mapped to the HAdV-D10 genome using minimap2 [62] to filter out host and positive control transcripts.

The mapped transcripts were then counted and classified, using a previously described script: “classify_transcripts_and_polya_segmented_V2.pl” [22]. These groups were then named using a second script: “name_transcripts_and_track.pl” [22] which utilises a table of features expected to be found in the HAdV-D10 genome and their expected transcription start site to produce a list of transcripts and which proteins they could produce if they were translated. Outputs were visualised in IGV [28–30] and graphs produced in GraphPad Prism version 10.4.1 (627) [63]. All scripts used for transcriptomic data analysis are available to access from Zenodo [64].

### Alignment of pVII transcripts

Transcript sequences for pVII on both the mature equivalent and precursor form were obtained from the dRNA-seq analysis output. They were aligned using Clustal Omega [31].

## Supporting information

S1 Fig Cryo-EM data of HAdV-D10 capsid. A Cryo-EM map of HAdV-D10 capsid at 3.3 Å resolution. B Example micrograph and 2D classes. In total 22,490 micrographs were collected at a nominal magnification of 105k. On average there is less than ~1 particle per micrograph with at least 11,344 particles in the dataset. C Fourier Shell Correlation (FSC) plots for Ewald sphere corrected map. D Example of model fitting to cryo-EM data with atomic model chain displayed in stick representation. Residues shown are penton 426–438. E Example of hexon model fitting to cryo-EM data, residues 646–655 and 919–928 are shown.
(DOCX)

S2 Fig Structure Of Capsid Minor Proteins. IX (panel A) forms a coiled-coil domain as observed in HAdV-C5 and HAdV-D26. Panel A displays contrasting ‘latching’ of hexon by the same IX molecule in HAdV-D10, latching hexon 3, compared to HAdV-C5 latching hexon 2. Despite the low particle number included in the final reconstruction the quality of the map density for some minor proteins is strong with many side chains modelled accurately particularly for IIIa (panel C). Panel D illustrates the VIII structure.
(DOCX)

S3 Fig Superimposition of HAdV-D10 for comparison to HAdV-C5 and HAdV-D26. ChimeraX’s MatchMaker command was used to first perform structural alignment and then calculate least-squares-fit root mean-square-deviations (RMSD) of Cα backbone residues. Colour scheme from Fig 1 for HAdV-D10 penton and hexon is maintained. A Comparison of HAdV-D10 penton, (cyan), and hexon (slate grey) with HAdV-C5. B Comparison of HAdV-D10 penton (cyan) and hexon (slate grey) with HAdV-D26.
(DOCX)

S4 Fig Local Resolution coloured Fiber Shaft Map and Fourier Shell Correlation of global fiber shaft resolution. Panel A fiber shaft map coloured by local resolution, the umbrella motif is resolved to 10 Å whilst the penton is resolved to around 4 Å. Panel B fourier shell correlation (FSC) plot for fiber shaft map with 0.143 cutoff illustrated with red line.
(DOCX)

S5 Fig Processing pipeline for focussed refinement of fiber shaft. Outline of single particle analysis processing performed in RELION 3.1 to achieve fiber shaft structure.
(DOCX)

S1 Table Cryo-EM data collection summary for capsid and fiber shaft maps including molecular modelling statistics for capsid. (DOCX)

S2 Table Per residue pLDDT scores for umbrella motif in HAdV-D10 fiber-penton (Fig 2) and fiber only (Fig 3) AlphaFold predictions. (DOCX)

S3 Table Percentage of each transcript produced across 24, 48 and 72 h.p.i. by cells infected with HAdV-D10. (DOCX)
