# Supplementary figures and images for: Identification of a novel fiber shaft structural motif and overexpression of key transcripts elucidated in human adenovirus D 10
Source: PLoS Pathog. 2026 Apr 28;22(4):e1014182. doi: 10.1371/journal.ppat.1014182 (PMC13148777; doi:10.1371/journal.ppat.1014182)

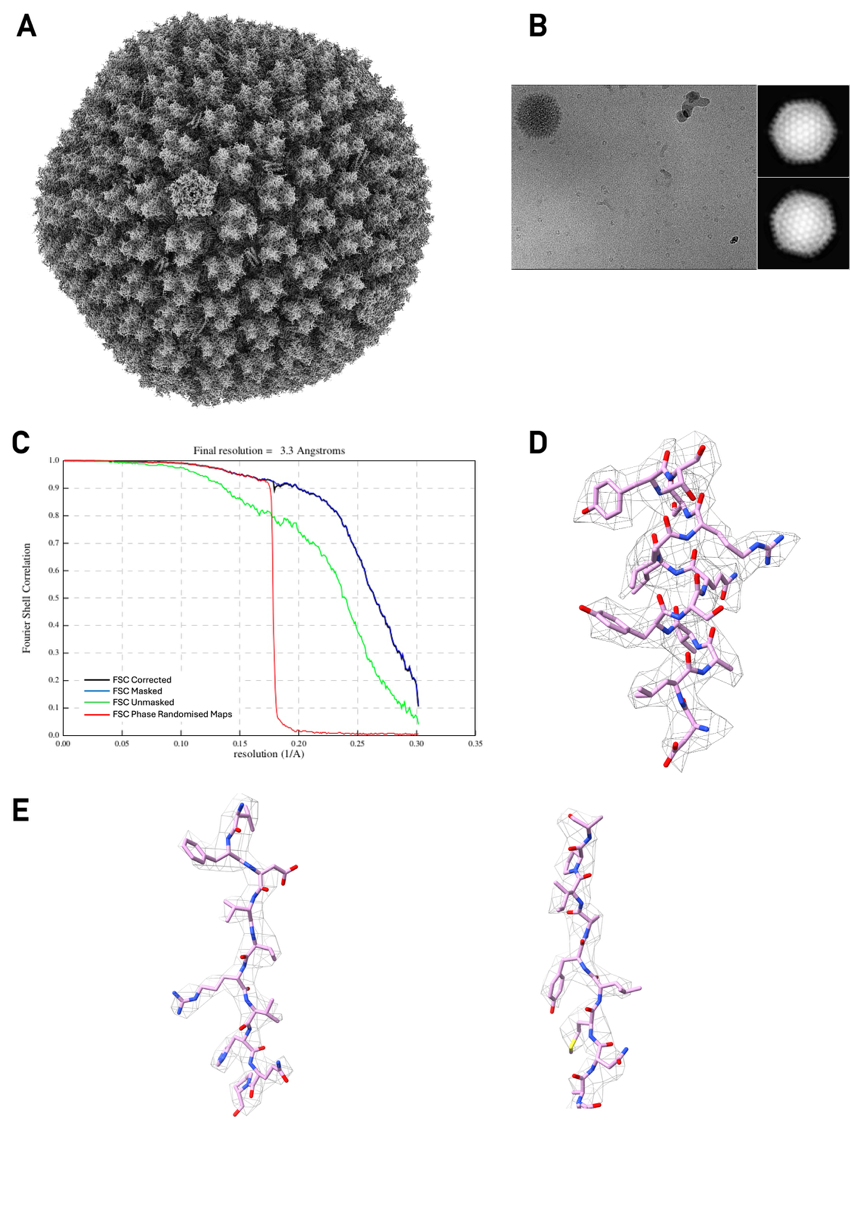


**S1 Fig**

Supplement: S1 Fig — A Cryo-EM map of HAdV-D10 capsid at 3.3 Å resolution. B Example micrograph and 2D classes. In total 22,490 micrographs were collected at a nominal magnification of 105k. On average there is less than ~1 particle per micrograph with at least 11,344 particles in the dataset. C Fourier Shell Correlation (FSC) plots for Ewald sphere corrected map. D Example of model fitting to cryo-EM data with atomic model chain displayed in stick representation. Residues shown are penton 426–438. E Example of hexon model fitting to cryo-EM data, residues 646–655 and 919–928 are shown. (DOCX) [file ppat.1014182.s001.docx]

**
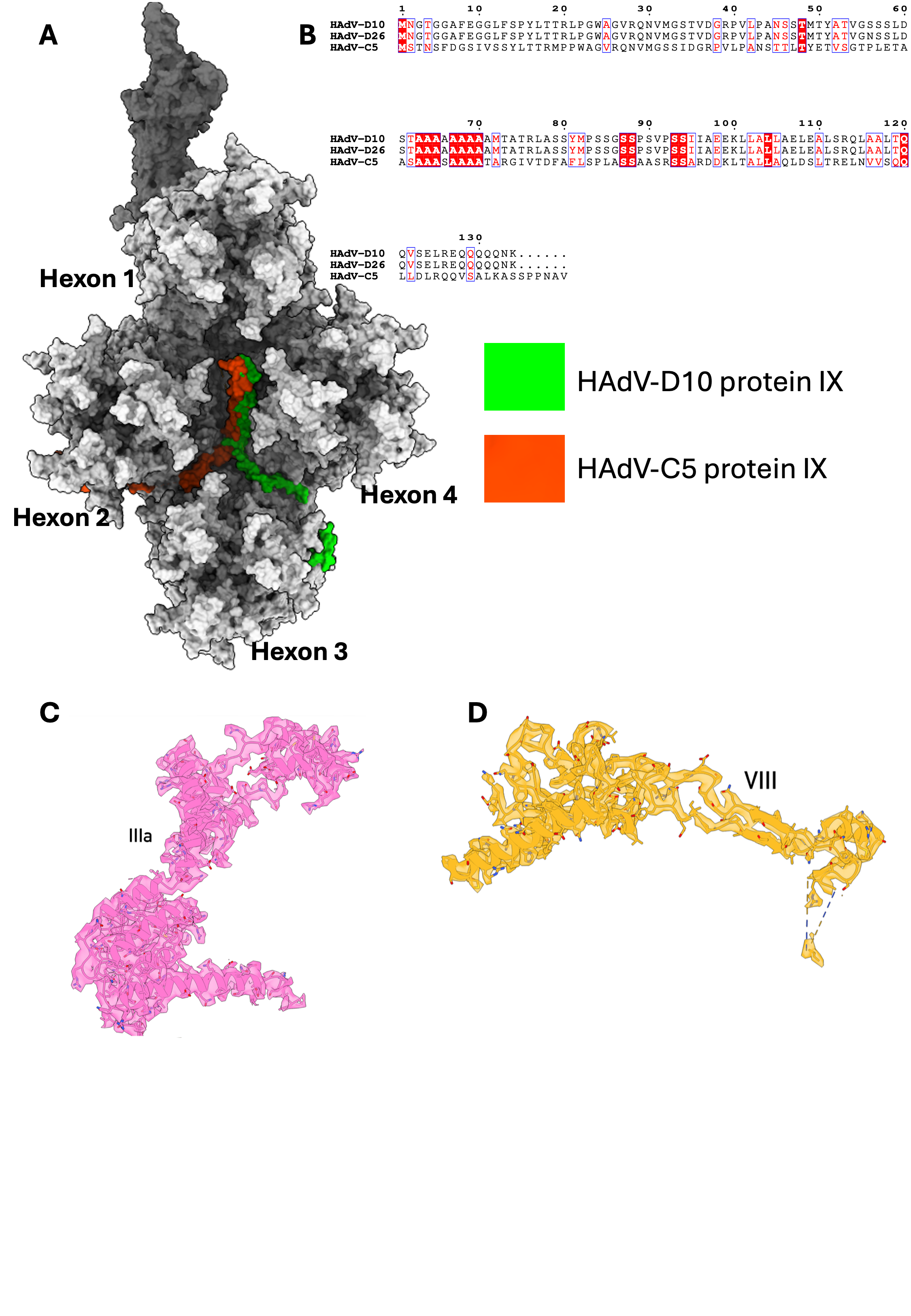
**

**S2 Fig**

Supplement: S2 Fig — IX (panel A) forms a coiled-coil domain as observed in HAdV-C5 and HAdV-D26. Panel A displays contrasting ‘latching’ of hexon by the same IX molecule in HAdV-D10, latching hexon 3, compared to HAdV-C5 latching hexon 2. Despite the low particle number included in the final reconstruction the quality of the map density for some minor proteins is strong with many side chains modelled accurately particularly for IIIa (panel C). Panel D illustrates the VIII structure. (DOCX) [file ppat.1014182.s002.docx]

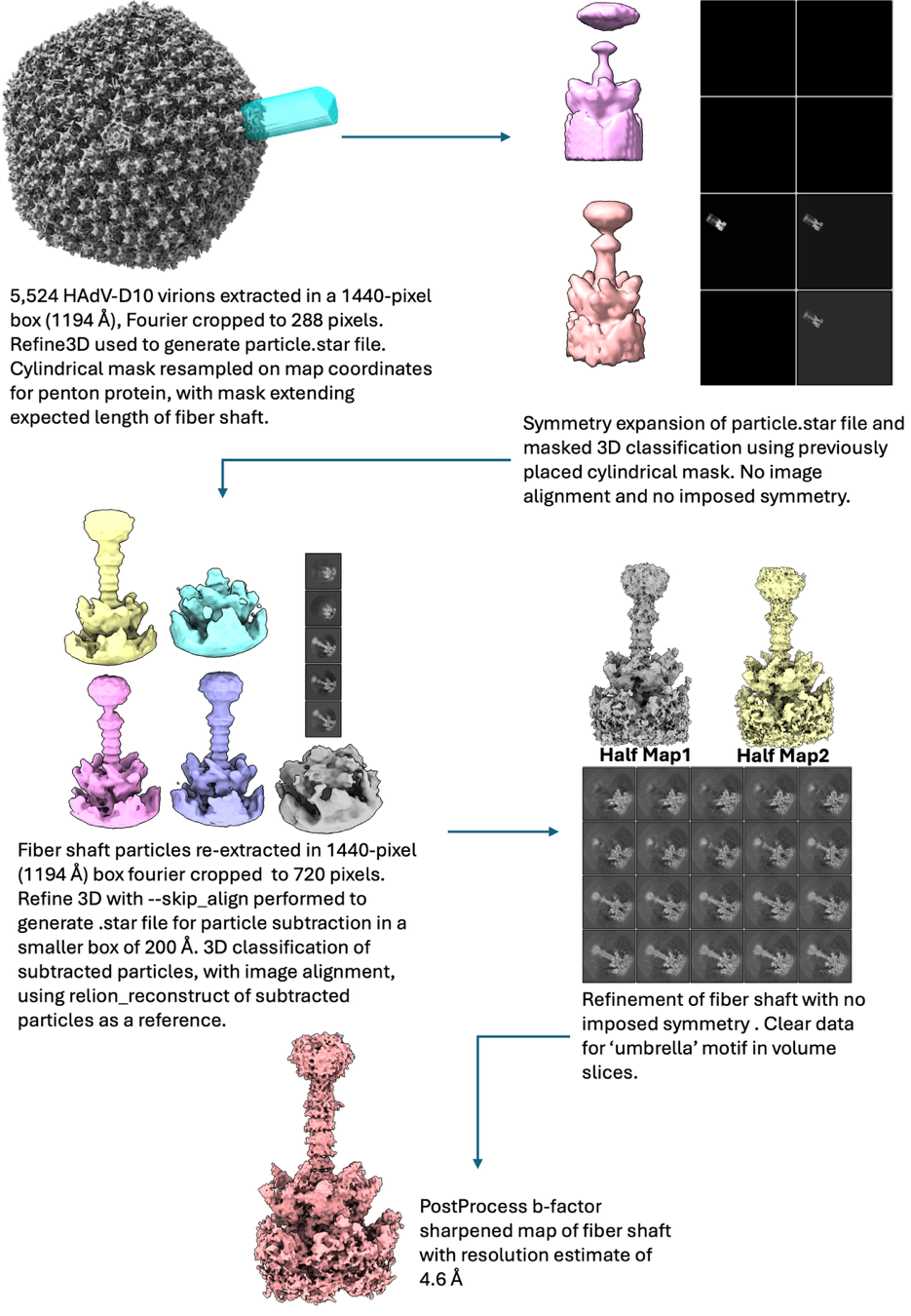


**S5 Fig**

Supplement: S5 Fig — Outline of single particle analysis processing performed in RELION 3.1 to achieve fiber shaft structure. (DOCX) [file ppat.1014182.s005.docx]
