# Supplementary material for: Identification of a novel fiber shaft structural motif and overexpression of key transcripts elucidated in human adenovirus D 10
Source: PLoS Pathog. 2026 Apr 28;22(4):e1014182. doi: 10.1371/journal.ppat.1014182 (PMC13148777; doi:10.1371/journal.ppat.1014182)
Supplement: S1 Table — (DOCX) [file ppat.1014182.s006.docx]

| **Microscope**  **S1 Table.** | Titan Krios |
| --- | --- |
| **Camera** | Gatan K3 |
| **Voltage (kV)** | 300 |
| **Grid Type** | Quantifoil Cu R2/2 + 3 nM Continuous Carbon Layer |
| **Grids Imaged** | 1 |
| **Magnification** | 105x |
| **Defocus Range (μm)** | 0.2 - 2.2 |
| **Pixel Size (Å)** | 0.829 |
| **Total Electron Dose e-/Å2** | 40 |
| **Number of frames** | 40 |
| **Number of Images** | 22490 |
| **Capsid** |  |
| **Initial Particle Number** | 11344 |
| **Final Particle Number** | 5524 |
| **Sampling Rate (Å/pixel)** | 1.658 |
| **Box Size (Å)** | 1193.76 1193.76 1193.76 |
| **Resolution (Å)** | 3.3 |
| **Symmetry** | I2 |
| **Map Sharpening B-factor** | 82.97 |
| **EMDB Deposition** | EMD-53736 |
| **PDB Deposition** | 9R78 |
| **Clashscore** | 1.18 |
| **Poor rotamers** | 171 (1.56%) |
| **Favoured rotamers** | 10399 (94.58%) |
| **Ramachandran outliers** | 99 (0.78%) |
| **Ramachandran favoured** | 11677 (92.14%) |
| **Rama distribution Z-score** | -1.88 ± 0.07 |
| **MolProbidity score** | 1.46 |
| **Cβ deviations >0.25 A** | 101 (0.85%) |
| **Clashscore** | 1.18 |
| **Poor rotamers** | 171 (1.56%) |
| **Favoured rotamers** | 10399 (94.58%) |
| **Ramachandran outliers** | 99 (0.78%) |
| **Ramachandran favoured** | 11677 (92.14%) |
| **Rama distribution Z-score** | -1.88 ± 0.07 |
| **MolProbidity score** | 1.46 |
| **Cβ deviations >0.25 A** | 101 (0.85%) |
| **Fiber Shaft** |  |
| **Box Size (Å)** | 200 200 200 |
| **Sampling Rate (Å/pixel)** | 1.658 |
| **Resolution (Å)** | 4.6 |
| **Symmetry** | C1 |
| **Map Sharpening B-factor** | 139.05 |
| **EMDB Deposition** | EMD-53655 |

**S1 Table. Cryo-EM data collection summary for capsid and fiber shaft maps including molecular modelling statistics for capsid.**
