# Supplementary material for: Identification of a novel fiber shaft structural motif and overexpression of key transcripts elucidated in human adenovirus D 10
Source: PLoS Pathog. 2026 Apr 28;22(4):e1014182. doi: 10.1371/journal.ppat.1014182 (PMC13148777; doi:10.1371/journal.ppat.1014182)
Supplement: S2 Table — (DOCX) [file ppat.1014182.s007.docx]

**S2 Table.**

| \| **Residue Sequence** \| **Residue Name** \| **HAdV-D10 Fiber-Penton Model pLDDT Mean** \| **HAdV-D10 Fiber Only plDDT Mean** \| \| --- \| --- \| --- \| --- \| \| 111 \| LYS \| 83 \| 85 \| \| 112 \| THR \| 88 \| 90 \| \| 113 \| GLY \| 87 \| 89 \| \| 114 \| ASN \| 85 \| 90 \| \| 115 \| GLY \| 89 \| 87 \| \| 116 \| LEU \| 90 \| 88 \| \| 117 \| LYS \| 84 \| 84 \| \| 118 \| VAL \| 87 \| 87 \| \| 119 \| ILE \| 85 \| 90 \| \| 120 \| ASP \| 79 \| 86 \| \| 121 \| LYS \| 71 \| 90 \| \| 122 \| SER \| 75 \| 87 \| \| 123 \| ILE \| 74 \| 82 \| \| 124 \| THR \| 73 \| 89 \| \| 125 \| ASP \| 65 \| 85 \| \| 126 \| LEU \| 65 \| 87 \| \| 127 \| PRO \| 69 \| 88 \| \| 128 \| GLY \| 78 \| 89 \| \| 129 \| LEU \| 76 \| 86 \| \| 130 \| ILE \| 80 \| 93 \| \| 131 \| ASP \| 86 \| 88 \| \| 132 \| THR \| 86 \| 94 \| \| 133 \| LEU \| 88 \| 88 \| \| 134 \| ALA \| 90 \| 90 \| \| 135 \| VAL \| 89 \| 88 \| \| 136 \| LEU \| 86 \| 89 \| \| 137 \| THR \| 89 \| 90 \| \| 138 \| GLY \| 90 \| 83 \| \| 139 \| LYS \| 86 \| 78 \| \| 140 \| GLY \| 93 \| 77 \| \| 141 \| ILE \| 92 \| 80 \| \| 142 \| GLY \| 92 \| 68 \| \| 143 \| THR \| 89 \| 77 \| \| 144 \| GLU \| 88 \| 73 \| \| 145 \| GLU \| 80 \| 73 \| \| 146 \| LEU \| 87 \| 66 \| \| 147 \| LYS \| 79 \| 70 \| \| 148 \| ASN \| 79 \| 66 \| \| 149 \| GLU \| 70 \| 66 \| \| 150 \| ASN \| 75 \| 68 \| \| 151 \| GLY \| 81 \| 65 \| \| 152 \| THR \| 81 \| 76 \| \| 153 \| ASN \| 78 \| 74 \| \| 154 \| LYS \| 79 \| 86 \| \| 155 \| GLY \| 87 \| 84 \| \| 156 \| VAL \| 87 \| 90 \| \| 157 \| GLY \| 89 \| 85 \| \| 158 \| LEU \| 92 \| 92 \| \| 159 \| ARG \| 85 \| 89 \| \| 160 \| VAL \| 94 \| 86 \| \| 161 \| ARG \| 86 \| 91 \| |
| --- | --- | --- | --- | --- | --- | --- | --- | --- | --- | --- | --- | --- | --- | --- | --- | --- | --- | --- | --- | --- | --- | --- | --- | --- | --- | --- | --- | --- | --- | --- | --- | --- | --- | --- | --- | --- | --- | --- | --- | --- | --- | --- | --- | --- | --- | --- | --- | --- | --- | --- | --- | --- | --- | --- | --- | --- | --- | --- | --- | --- | --- | --- | --- | --- | --- | --- | --- | --- | --- | --- | --- | --- | --- | --- | --- | --- | --- | --- | --- | --- | --- | --- | --- | --- | --- | --- | --- | --- | --- | --- | --- | --- | --- | --- | --- | --- | --- | --- | --- | --- | --- | --- | --- | --- | --- | --- | --- | --- | --- | --- | --- | --- | --- | --- | --- | --- | --- | --- | --- | --- | --- | --- | --- | --- | --- | --- | --- | --- | --- | --- | --- | --- | --- | --- | --- | --- | --- | --- | --- | --- | --- | --- | --- | --- | --- | --- | --- | --- | --- | --- | --- | --- | --- | --- | --- | --- | --- | --- | --- | --- | --- | --- | --- | --- | --- | --- | --- | --- | --- | --- | --- | --- | --- | --- | --- | --- | --- | --- | --- | --- | --- | --- | --- | --- | --- | --- | --- | --- | --- | --- | --- | --- | --- | --- | --- | --- | --- | --- | --- | --- | --- | --- | --- | --- | --- | --- | --- | --- |

**S2 Table. Per residue pLDDT scores for umbrella motif in HAdV-D10 fiber-penton (Fig2) and fiber only (Fig3) AlphaFold predictions.**
