# Supplementary material for: Identification of a novel fiber shaft structural motif and overexpression of key transcripts elucidated in human adenovirus D 10
Source: PLoS Pathog. 2026 Apr 28;22(4):e1014182. doi: 10.1371/journal.ppat.1014182 (PMC13148777; doi:10.1371/journal.ppat.1014182)
Supplement: S3 Table — (DOCX) [file ppat.1014182.s008.docx]

| Feature  **S3 Table.** | Percent of total (24hr) | Percent of total (48hr) | Percent of total (72hr) |
| --- | --- | --- | --- |
| E1a_12S | 0.917 | 0.0516 | 0.0521 |
| E1a_13S | 0.6986 | 0.0478 | 0.0678 |
| E1a_14.8K | 0.0436 | 0.0151 | 0.0157 |
| E1b_19K(smallT) | 6.6375 | 1.7743 | 1.3861 |
| E1b_55K | 0 | 0.0012 | 0 |
| E1b_largeTantigen | 0 | 0 | 0 |
| E2_DBP | 11.703 | 2.5064 | 1.1851 |
| E2_DNA_polymerase | 0 | 0 | 0 |
| E2_preTP | 0 | 0.005 | 0.0041 |
| E3_12.5kDa | 1.6157 | 0.9161 | 0.7542 |
| E3_14.6kDa_controlprotein | 0.262 | 0.3616 | 0.406 |
| E3_29.2K_CR1-gamma | 0 | 0.0315 | 0.0306 |
| E3_55kDa | 0 | 0 | 0 |
| E3_7.6KDa | 0 | 0 | 0 |
| E3_CR1-alpha | 0 | 0 | 0.0008 |
| E3_CR1-beta | 0.0873 | 0.0831 | 0.1273 |
| E3_CR1-gamma | 0 | 0 | 0 |
| E3_gp18.5k_19K_Glycoprotein | 0 | 0.0025 | 0 |
| E3_RID-alpha | 0 | 0 | 0 |
| E3_RID-beta | 0 | 0 | 0.0016 |
| E3_TSS | 0 | 0 | 0 |
| E4_34kDa_controlprotein | 0 | 0 | 0 |
| E4_ORF1protein | 1.048 | 0.0919 | 0.0719 |
| E4_ORF2protein | 0.0436 | 0.0201 | 0.0124 |
| E4_ORF3_protein | 0.9606 | 0.0655 | 0.0504 |
| E4_ORF4_protein | 0.262 | 0.0466 | 0.0396 |
| E4_ORF5protein | 0 | 0 | 0 |
| E4_ORF6_7_protein | 0 | 0 | 0 |
| E4_TSS | 0 | 0 | 0 |
| i-leader_protein | 1.3973 | 1.5575 | 1.1239 |
| L1_52kDa | 2.2707 | 3.2234 | 2.8003 |
| L1_IIIa | 0.917 | 1.7869 | 1.6375 |
| L2_penton | 1.572 | 2.7622 | 2.974 |
| L2_preMu_pXprecursor | 6.2445 | 13.6298 | 14.0505 |
| L2_preVII | 6.2882 | 13.6513 | 14.6634 |
| L2_pV | 1.6157 | 3.8119 | 4.7902 |
| L2_VII_bysplicing | 1.2663 | 2.6186 | 3.3983 |
| L3_hexon | 4.3231 | 7.6453 | 9.1065 |
| L3_preVI | 2.4017 | 2.3464 | 3.1816 |
| L3_protease | 0.2183 | 1.1971 | 1.171 |
| L4_100kDa | 4.8908 | 10.2728 | 6.6585 |
| L4_22kDa_full | 0.4803 | 0.2847 | 0.1695 |
| L4_33K | 4.1484 | 4.8364 | 3.3784 |
| L4_33K_alt_splice | 0.131 | 0.0995 | 0.0686 |
| L4_pVIII | 1.6157 | 3.2902 | 4.6769 |
| L5_fiber | 1.0917 | 2.1838 | 2.2024 |
| none from list | 8.6462 | 6.9913 | 6.1829 |
| pIVa2 | 2.096 | 2.0654 | 2.3636 |
| pIX | 12.5764 | 6.4721 | 7.7832 |
| UXP | 0 | 0 | 0 |

**S3 Table. Percentage of each transcript produced across 24, 48 and 72 h.p.i. by cells infected with HAdV-D10.**
